# New Mathematical Model for Correlation Between Tensile Elastic Modulus and Shore “A” and “00” Hardness for Flexible Polymers

**DOI:** 10.3390/polym18050620

**Published:** 2026-03-01

**Authors:** Josip Hoster, Nikola Šimunić, Tihana Kostadin, Bruno Vojnović

**Affiliations:** Mechanical Engineering Department, Karlovac University of Applied Sciences, Trg J.J. Strossmayera 9, 47000 Karlovac, Croatia; nikola.simunic@vuka.hr (N.Š.); tihana.kostadin@vuka.hr (T.K.); bruno.vojnovic@vuka.hr (B.V.)

**Keywords:** flexible polymers, Shore hardness, initial elastic modulus, hyperelastic, correlation, exponential function, rational function, true stress, logarithmic strain

## Abstract

The paper presents the development of a correlation model for initial tensile elastic modulus for flexible polymers as a function of Shore hardness in OO and A scale based on measurement. Measured polymers are in groups of silicone rubber, nitrile butadiene rubber (NBR), thermoplastic polyurethane (TPU) and silicone. The model is composed of piecewise exponential functions with fixed coefficients chosen to minimize the S_2_ error norm and absolute value of relative error at the measured data points. Every chosen section of the hardness scale has one exponential function correlating the hardness to tensile elastic modulus with the argument in the form of a polynomial up to the fourth degree. The coefficients for the polynomial arguments were determined by enforcing interpolation conditions in a chosen set of points in the logarithmic scale for the elastic modulus. The correlation model possesses C0 continuity. For each material, five specimens were used for hardness measurements and five for the elastic modulus testing. The correlation model gives a positive value for elastic modulus of 0 for hardness, and a “finite”, “reasonable” value of 100 for hardness and is monotonic. Tensile properties were evaluated using true stress and logarithmic (Hencky) strain, with iterative correction of the changing cross-sectional area to account for large strain. The maximum relative error achieved in the correlation model for the OO scale is 13.4%, while for the A scale it is 7%. The developed model provides a practical and rapid method for estimating the initial tensile elastic modulus from non-destructive hardness measurements and is particularly useful in industrial applications and in the development of material models for dental surgery simulations.

## 1. Introduction

This paper presents the development of a new mathematical model for the correlation between the initial tensile elastic modulus of flexible polymers and Shore hardness on the OO and A scales. The developed model(s) (one for the A scale, and the other for the OO scale) offer reliable correlation for the elastic tensile modulus under 100 Shore A hardness and above 0 Shore OO hardness. Hardness measurement is much cheaper and quicker than the measurement of tensile properties; therefore, a reliable correlation between hardness and elastic modulus offers the advantage of saving time and money in some practical applications. Hardness measuring is a non-destructive measurement that can be conducted quickly on a part, product, or material specimen, using a hand-held durometer, whereas tensile testing is performed only on a prepared specimen in a tensile testing machine. Fast hardness measurement with a reliable correlation to the elastic modulus can save money and material, which is pronounced in industry and additive production.

Reliable data for the correlation model were obtained by measuring hardness according to ISO 48-4:2018 [1] and ASTM–D2240 [2], and tensile properties according to ISO 37: 2017 [3], with logarithmic strain [4,5] and the current cross-section for stress determination. Specimens for tensile testing comply with ISO 37:2017, and specimens for hardness testing comply with ISO 48-4:2018 and ASTM2240. With the acquired data, the best suited function [6] for approximation of elastic modulus was tested and developed from the set of polynomial, rational and exponential functions which meet all the criteria set to satisfy this contribution. All hardness measurements were conducted on the specimen’s surface that was exposed to air during the curing process, considering the possible difference in hardness on the air and container interface [7], which those authors reported not to exceed 2.5 units.

The data for the elastic modulus were obtained using machine tensile testing. A detailed description of how each stress–strain diagram was created is shown in the manuscript. The details of correlation functions will be presented.

Three types of correlation functions were considered and analyzed for the best suited one, namely polynomial, rational and exponential. The polynomial function was tested and defined on the entire hardness scale and as a piecewise function with different degrees. In the rational function, the numerator and denominator were tested with various degrees, defined over the entire hardness scale. An exponential function was used in two forms: as a sum of exponential functions, each with its own parameter in the argument, defined over the entire hardness scale; and as a piecewise function, with its argument expressed as a polynomial, and also defined piecewise.

The applicability of the proposed equations was established by approximating data derived from five test specimens for each flexible polymer, taking the mean value of both hardness and the initial tensile elastic modulus. From the scatter of the tensile data combined with the uncertainty of the cross-section measurements, the upper and lower limits for a correlated value at a measured point were calculated. The materials tested include silicone rubbers with a declared hardness of 15, 20 and 30 Shore A; nitrile butadiene rubber (NBR) with 40 and 45 Shore A; silicone 60 Shore A; and thermoplastic polyurethane (TPU) with 60 A, 70 A, 85 A and 98 A. Polymers included in both scales, A and OO, are all listed except for TPU 98 A, which exceeds 100 on the OO scale. The silicone rubber specimens were manufactured by molding, while TPU test specimens were produced using additive technology. Other specimens were cut out of sheets obtained from the supplier, according to ISO 23529:2016 [8]. The tensile testing conditions were a 500 mm/min displacement rate, an air temperature of 22 °C and 45% RH. Five specimens were used for each material.

Thickness measurements of the specimens were performed according to ISO 23529:2016, while the measurement of length and cross-section width was performed by a digital caliper, with an accuracy of ±0.05 mm. Tensile testing was performed on a SHIMADZU AG-X tensile testing machine, with class 1 displacement measuring capability. The change in the cross-section’s dimensions, or in other words, Poisson’s ratio, was measured for each material on a strip-shaped specimen. This relationship of transversal strain to the longitudinal strain was modeled by means of interpolation with polynomials. It was used for the approximation of the true cross-sectional area, therefore, the true stress for each given force. The strain was calculated using a logarithmic equation, according to Hencky [4,5], chosen for its additive property, as opposed to the engineering strain.

The hardness measurement was conducted on a Shore OO durometer, according to ASTM-D2240, and a Shore A durometer, according to ISO 48-4:2018. Many of the tested materials showed pronounced hyperelastic behavior derived from the stress, calculated as the current force divided by the current cross-sectional area and logarithmic strain. Only the initial elastic modulus is used in the correlation model; not all the parameters are necessary to describe the stress–strain curve for the large strain and not the “secant” [9] modulus.

In all the approximation functions, the first criterion for acceptance for a correlation function is non-negativity, i.e., the elastic modulus calculated for a hardness value of 0 on each scale must not give zero modulus and rather a small positive value. The second criterion is the value for the modulus for a hardness value of 100 in each scale which must be either a specific (known) measured number, or, if it is not obtainable, a “good” estimate based on the trend of approximated measured values below on the scale, or the value from the higher Shore scale, according to the correlation between the scales.

This work represents a first step in a project focused on identifying suitable materials for dental surgery models, where mechanical properties are critical for their intended application. Rapid characterization of these materials through hardness measurements is particularly valuable in terms of reducing both time and cost. The immediate next step of this research involves acquiring additional materials with hardness values close to those of biological tissues in the human oral cavity and refining the developed correlation models. Future work will focus on detailed hardness measurements on the OO scale, as well as on the comprehensive characterization of the mechanical properties of these tissues.

## 2. Materials and Methods

The aim of this manuscript is to determine the correlation parameters between the Shore hardness measurement data, limited to the OO and A scales, and the initial tensile elastic modulus derived from the single-axis tensile measurement of flexible polymers. The stress–strain curve is strongly nonlinear for most of the tested polymers; therefore, the initial elastic modulus, i.e., the slope of the stress–strain curve at 0 strain, is the selected value for the correlation.

**Figure 1:**
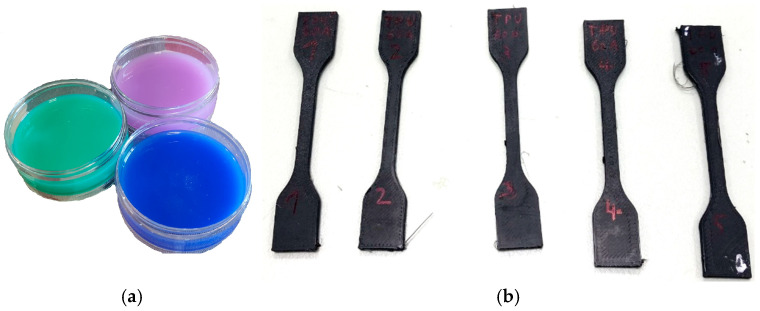
Samples made from flexible polymers: (**a**) ISO 868 hardness measurement from silicone rubbers, (**b**) ISO 37:2017 TPU 70A for tensile measurement.

As the first step, measurements of hardness of flexible polymers on samples produced according to ISO 48-4:2018, ASTM-D2240 and ISO 868, shown on Figure 1a, were performed. As the second step, the samples for tensile testing according to ISO 37:2017 were produced, shown in Figure 1b. Figure 1a shows silicone rubbers of declared 15, 20, and 30 Shore A hardness, while Figure 1b shows specimens of TPU 70A produced using the FDM technology. The intention is to test materials which can be measured in both hardness scales.

### 2.1. Tensile Testing—The Elastic Modulus

**Figure 2:**
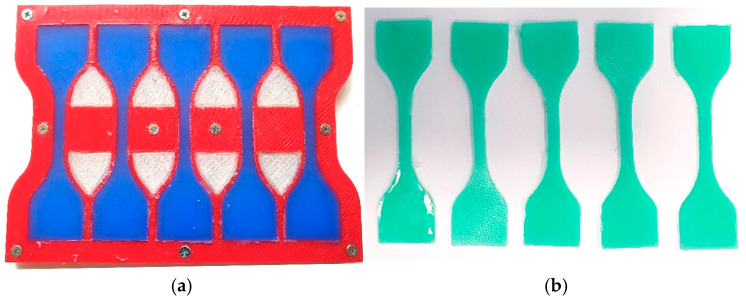
The mold and specimens from silicone rubber: (**a**) molded specimens with 30 Shore A, (**b**) specimens with 15 Shore A.

The elastic modulus for the materials in this study is calculated from the test results from the tensile testing machine SHIMADZU AG-X (Shimadzu, Kyoto, Japan) on specimens shaped according to ISO 37:2017. The specimens from thermoplastic polyurethane were manufactured using the additive technology, and are shown in Figure 1b. The specimens for silicone rubbers (Quanzhou HengChao Technology Co. Ltd., Quanzhou, China), shown in Figure 2, were produced using molds. Since the change in geometry of specimens is significant, the change in the cross-section (shrinkage) was taken into account. 

Silicone rubbers, commercially available, were used as one group of flexible polymers, tested according to ISO 37:2017. The specimens have a thickness of 2^±0.15^ mm, and were molded in a polymer mold as shown in Figure 2. The mold was manufactured using FDM technology.

**Figure 3:**
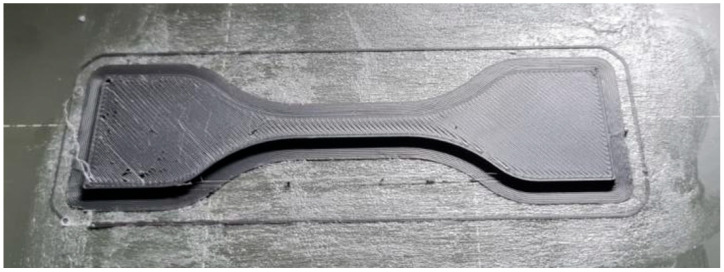
TPU 60A specimen after FDM printing.

Test specimens from TPU materials were manufactured by FDM technology, while NBR (Gumiimpex d.o.o., Varaždin, Croatia) specimens had to be cut from available plates. In Figure 3, a TPU specimen is shown after manufacturing.

Since the change in geometry is large, the engineering strain is not appropriate to show the detailed behavior of such materials; therefore, the logarithmic strain is chosen to represent the change in geometry. It possesses the additive property [4]. Here is a brief description of how the logarithmic strain is calculated from the current length of the test specimen, or a part of it, and the initial length of that part. The differential of the strain is calculated from dε=dl/l. Integrating the contribution of strain differentials from the initial length to the current one gives:

(1) $$\varepsilon =\int_{l_{0}}^{l_{i}}d\varepsilon =\int_{l_{0}}^{l_{i}}dl/l=\ln{\left.\left(l\right)\right|}_{l_{0}}^{l_{i}}=\ln\left(l_{i}\right)-\ln\left(l_{0}\right)=\ln\left(\frac{l_{i}}{l_{0}}\right).$$

**Figure 4:**
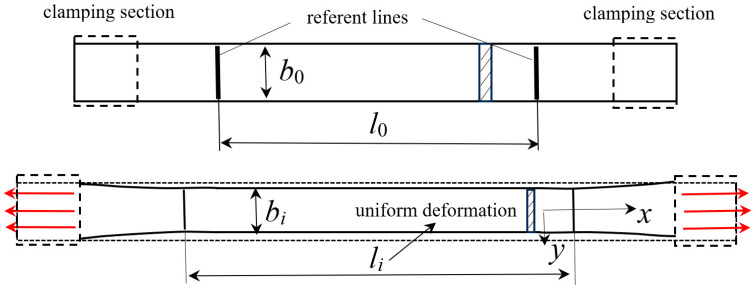
The tensile shrinkage testing.

**Figure 5:**
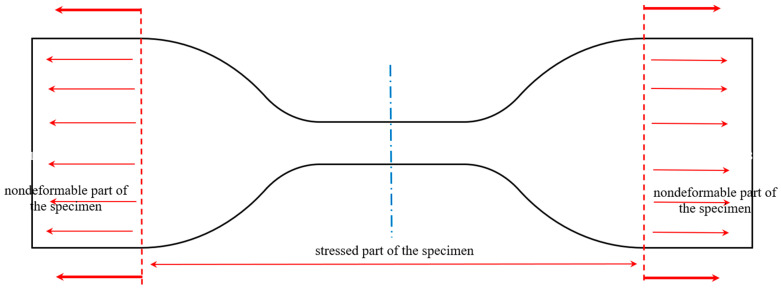
The tensile testing specimen according to ISO 37:2017.

For the purpose of taking into account the change in the cross-section, measurements of strip-shaped specimens were carried out. One of these specimens is symbolically shown in Figure 4. The part of the specimen that has uniform deformation was chosen as the measuring representative part, and 2 perpendicular referent lines were marked on the edges of that area. The part of the specimen with uniform deformation was determined after an initial stretch to the same strain experienced by the tensile testing specimens; one is shown in Figure 5. The details of the strip-shaped specimens are shown in the lower section of Figure 4. Then, for several values of force, the longitudinal displacement of the referent lines and perpendicular edges were measured, from which the logarithmic strain was calculated according to Equation (1). After the measurement was carried out, an approximation of the perpendicular strain as a function of the longitudinal one for the tested material was interpolated by polynomial function in the form of:

(2) $$\varepsilon_{y}=\sum_{i=1}^{3}a_{i}{\left(\varepsilon_{x}\right)}^{i}.$$

For each material, a set of coefficients *a*_i_ was derived by solving a system of equations arriving from the interpolation conditions. Those coefficients were used in the calculation of the stress–strain diagram for a given material. The function (2) was used in an iterative procedure of calculating the stress–strain relationship for tensile testing specimens such as the one in Figure 3.

In Figure 4, the tensile shrinkage testing is shown symbolically. The test specimens used in such tests are strips with a rectangular cross-section. Initial width and thickness are denoted as *b*_0_ and *t*_0_, respectively. The data resulting from the testing in the tensile testing machine SHIMADZU AG-X is a set of force-displacement data, *u*, of the clamping chuck. Since the displacement means extension of the non-constant cross-section in the case of specimens in Figure 5, the first step is to connect displacement and force to the strain and stress, following the change in the cross-section geometry according to Equation (2).

**Figure 6:**
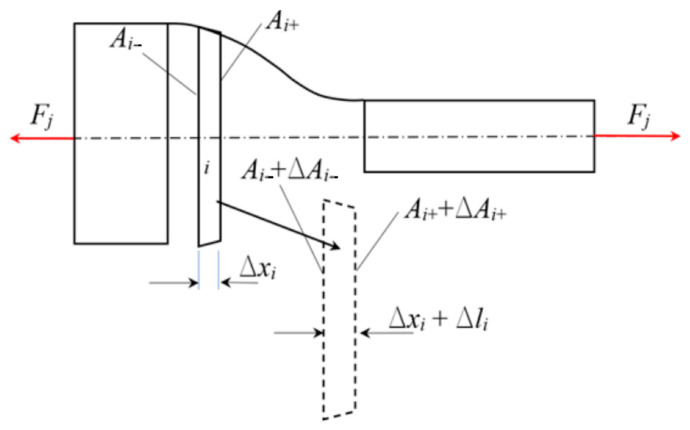
The tensile testing specimen—stressed and deformed section.

Measurements of change in length and cross-section’s measures were performed using a digital caliper. In Figure 5, a tensile specimen according to ISO 37:2017 [3] is shown, with its specific regions, i.e., the clamped part, the wide rectangular part at the ends, and the stressed part, in between. Since the specimen shown in Figure 5 has a variable cross-section, the correlation between the displacement and strain shall be calculated numerically using finite segments of the specimen, shown symbolically in Figure 6.

The elastic modulus will be determined iteratively until the summated change in length of all the sections gives the overall change in length, i.e., the clamping chuck displacement, here denoted *u*. In Figure 5, that is the change in length of the section denoted as “stressed part of the specimen”. The details of the geometry of each specimen segment are shown in Figure 6.

Calculating the change in length of the stressed part of the specimen starts by reading the value of the force *F_j_* from the diagram. For each segment of the specimen, the mean value of the cross-sectional area, *A_i_,* is calculated as the mean value of *A_i_*_−_ and *A_i_*_+_. With that data, the mean value of the stress s^(1)^ is calculated. Here, the upper index in parenthesis stands for the first iteration. In the second step, the strain from the stress and an initial guess of the elastic modulus, *E*_(0)_, is calculated. In the third step, the elongation of the segment D*x_i_* is calculated, using equation $\Delta l_{i}\approx F_{j}\Delta x_{i}/\left(A_{i}E_{\left(0\right)}\right).$ In the fourth step, the change in the cross-section, therefore the new cross-section, *A_i_*_+1_, is calculated using Equation (2), and the procedure from the first step with the updated data is repeated. The change in length for the stressed part of the specimen is the summated changes in length of all segments in the *k*-th iteration depicted in the following equation:

(3) $$\Delta l^{\left(k\right)}=\sum_{i=1}^{n}\Delta l_{i}=\sum_{i=1}^{n}\frac{F_{j}\Delta x_{i}}{A_{i}E_{\left(k\right)}}=u^{\left(k\right)}.$$

The procedure is repeated until the change in length of the stressed part of the specimen in the current iteration has a value less than 5% different from the previous iteration, i.e., $u_{\left(k+1\right)}/u_{\left(k\right)}\leq 1.05$. The value 1.05 is used because the convergence has proven monotonic for all the materials and force values, from “below”.

At least two steps of corrections were conducted for all the force values and for all the tested materials in order to gain satisfactory convergence of displacement *u*. Written symbolically, the algorithm for displacement *u* for a given force follows the form:

(4) $$\Delta l^{\left(1\right)}=u^{\left(1\right)}=\sum_{i=1}^{n}\frac{F_{j}\Delta x_{i}}{A_{i}^{\left(0\right)}E^{\left(1\right)}}.\;\Rightarrow \Delta l^{\left(2\right)}=u^{\left(2\right)}=\sum_{i=1}^{n}\frac{F_{j}\Delta x_{i}}{A_{i}^{\left(1\right)}\left(\varepsilon^{\left(0\right)}\right)E^{\left(2\right)}}.\;\Rightarrow \Delta l^{\left(3\right)}=u^{\left(3\right)}=\sum_{i=1}^{n}\frac{F_{j}\Delta x_{i}}{A_{i}^{\left(2\right)}\left(\varepsilon^{\left(1\right)}\right)E^{\left(3\right)}}\ldots$$

The maximum stress in the narrowest (measuring) part of the specimen will be calculated according to equation:

(5) $$\sigma^{\left(n\right)}=\frac{F_{j}}{A_{\min}^{\left(n\right)}}.$$

**Figure 7:**
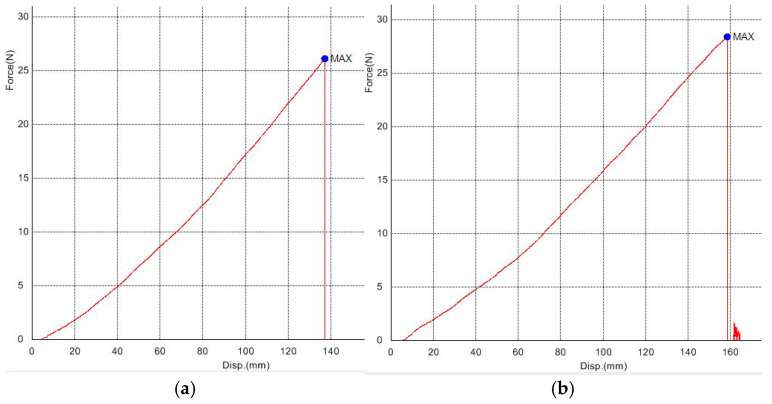
Shimadzu AG-X output for silicone rubber with 15 Shore A: (**a**) the first of five samples, (**b**) the second of five samples.

**Figure 8:**
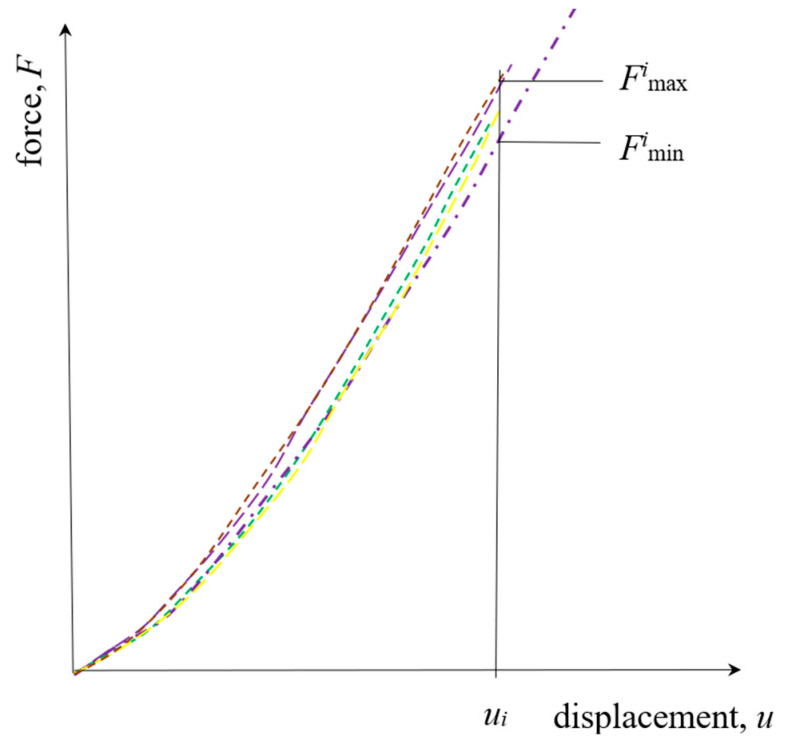
Force-displacement curves scatter for silicone rubber with 30 Shore A.

Here, the $A_{\min}^{\left(n\right)}$ is the converged cross-section of the measuring part of the specimen. Some of the force-displacement diagrams from the tensile testing machine are shown in Figure 7. Diagrams in Figure 7, and all the others for all the other materials, need some editing before using them in calculation. Firstly, shifting the 0.0 values for the curve start, mostly because of the fluctuating start section and similar reasons. Then, several values from the curves were read (extracted) as a function of displacement. The mean value of the curves for five samples was taken as representation of behavior for that material. In Figure 8, all 5 force-displacement curves for silicone rubber with 30 Shore A are shown in a diagram, depicting dissipation or scatter. The diagram is in scale, without specific numbers shown. This material was selected as the one with the most dissipative curves of all the measured materials. The maximum difference for this material, i.e., the ratio of the minimum to the maximum force for the maximum displacement all five specimens have endured, is 0.895. This yields a maximum scatter of +/−5.5% w.r.t. the mean value.

**Figure 9:**
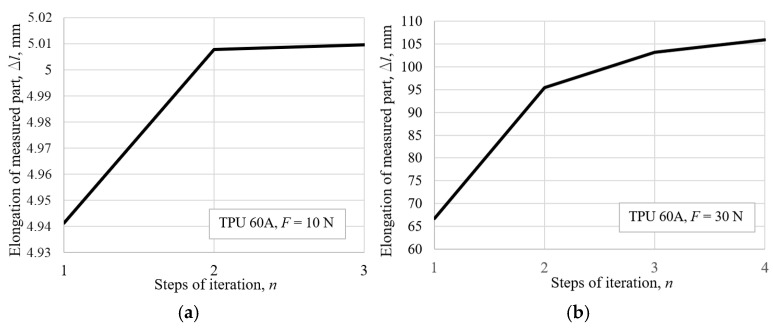
The TPU 60A tensile testing specimen’s convergence of elongation: (**a**) for *F* = 10 N, (**b**) for *F* = 30 N.

With the data prepared in the way described above, the iterative procedure detailed in Equations (4) and (5) was conducted. After performing the combined calculations based on the measured data, the convergence of displacement (elongation) for TPU 60A is illustrated in Figure 9. For most of the materials, three steps were required for a satisfactory convergence in most of the force range.

In Figure 9b, the pronounced nonlinearity in convergence of displacement through the calculation steps is evident for the higher strain, compared to the convergence rate for the lower force values in Figure 9a. The greater the change in geometry, i.e., the greater the acting force, the more pronounced nonlinear behavior the material exhibits; therefore, more steps in correction of the stress–strain curve, or elongation of the specimen, are needed. Expected reliability of measurement is 0.1 mm, i.e., +/−0.05 mm from the measured value. During the tensile testing, the width, *b*_meas_, of the specimen was measured with a digital caliper, while the thickness *t*_approx_ was approximated from the current width by using the polynomial correlation of the longitudinal to lateral strain, according to Equation (2). This leads to uncertainty, i.e., the upper and lower limits, of the cross-sectional area calculated as:

(6) $$\begin{array}{l} A_{\min}=\left(b_{\mathrm{meas}}-0.05\right)\left(t_{\mathrm{approx}}\right)\;{\mathrm{mm}}^{2}.\\ A_{\max}=\left(b_{\mathrm{meas}}+0.05\right)\left(t_{\mathrm{approx}}\right)\;{\mathrm{mm}}^{2}. \end{array}$$

Since the initial slope on the stress–strain curve was correlated, the uncertainty was calculated for the first three increments of force. This yields the scatter in an amount of +/−2.5% w.r.t. the mean value. Combined with the scatter of the force as a function of displacement, the scatter for the tensile elastic modulus is expected to be within the limits of +/−8% w.r.t. the mean value shown in the diagrams.

### 2.2. The Results of Stress–Strain Calculation

**Figure 10:**
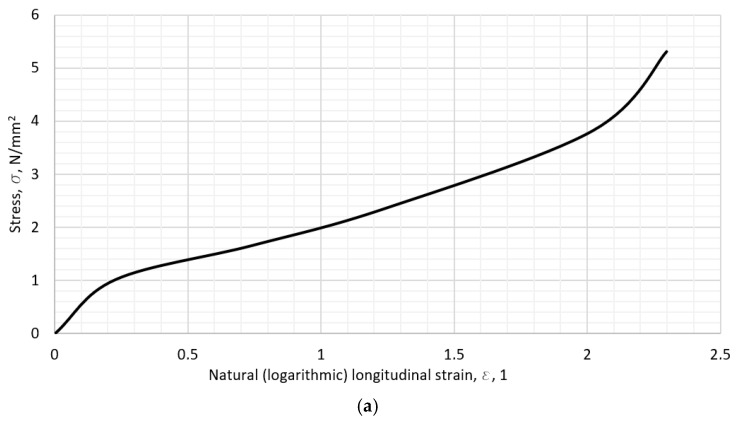

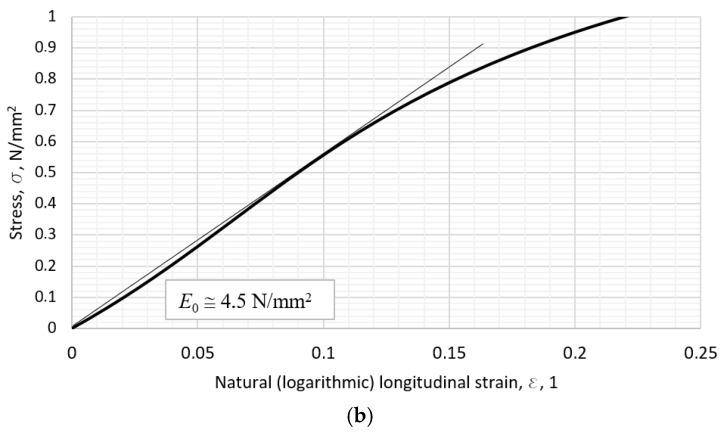
The TPU 60 stress–strain diagram: (**a**) the overall calculated curve, (**b**) the initial elastic modulus (slope).

**Figure 11:**
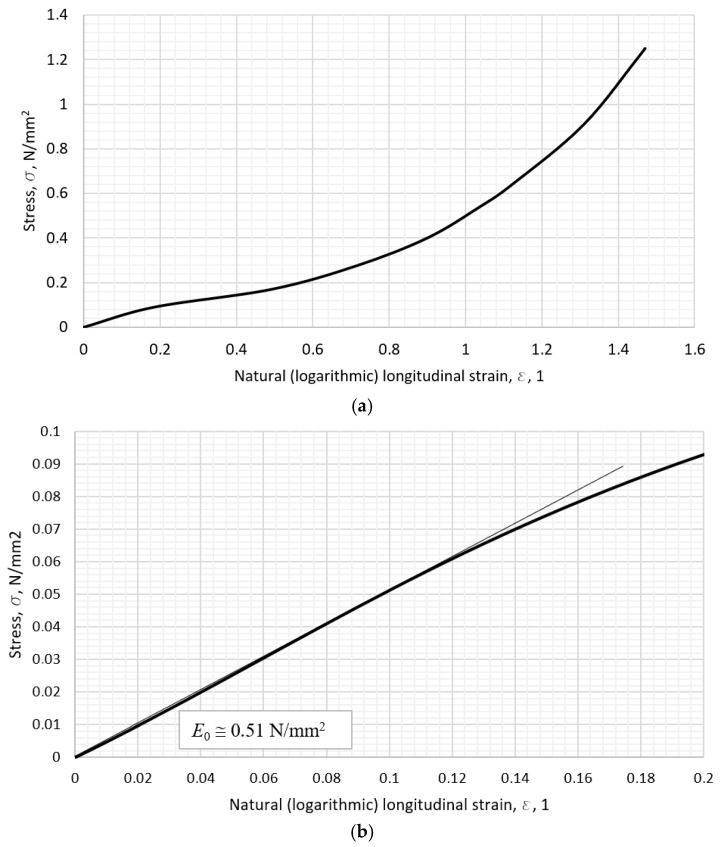
The Silicone rubber 30 A stress–strain diagram: (**a**) the overall calculated curve, (**b**) the initial elastic modulus (slope).

Most of the materials show pronounced hyperelastic behavior, such as TPU 60A and Silicone rubber 30 A, with their stress–strain curves shown in Figure 10 and Figure 11. On Figure 10b and Figure 11b, the first section of the curve is amplified in order to emphasize the data chosen for the representation of the given material. The initial slope on the stress–strain curve is the chosen value of the modulus in the correlation model further on. The value of the initial slope for each material’s curve was calculated graphically, i.e., a tangent line was drawn against the curve that best fits it, and the value of tangent written as the initial slope—the tensile modulus. The initial slope is shown by the thin line in Figure 10b and Figure 11b. 

### 2.3. Hardness Shore A and OO Testing

**Figure 12:**
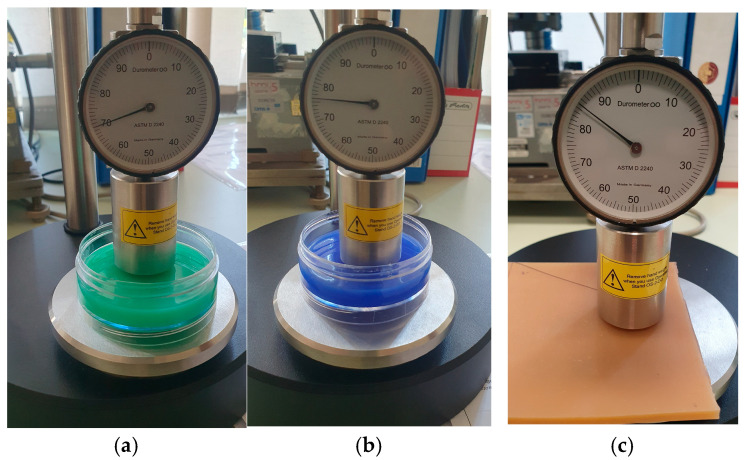
Shore OO scale hardness measurement: (**a**) Silicone rubber 15 A, (**b**) Silicone rubber 30 A, (**c**) natural rubber 40 A.

**Figure 13:**
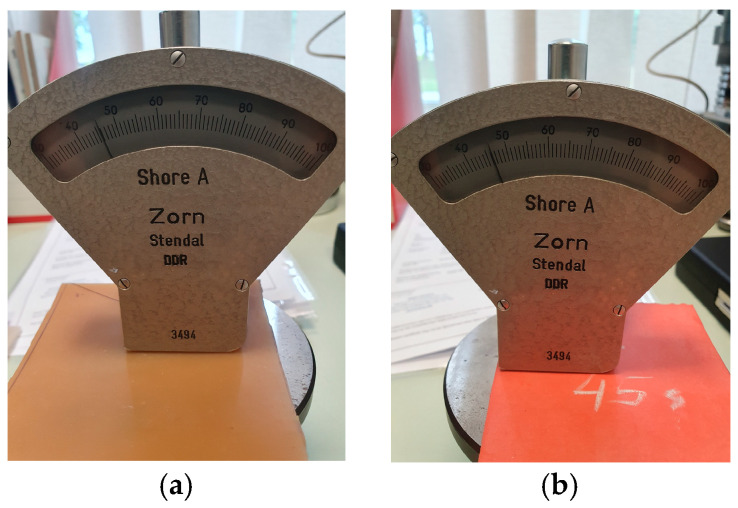
Shore A scale hardness measurement: (**a**) natural rubber 40 A, (**b**) natural rubber 45 A.

All the materials used in this study were tested on Shore hardness testers, for the Shore OO scale, according to ASTM 2240 [2], and for the Shore A scale a tester manufactured by Zorn Stendal, former DDR. A few specimens tested on the Shore OO hardness tester are shown in Figure 12. Some specimens tested on the Shore A tester are shown in Figure 13. The durometers available in our laboratory have a Shore A scale ranging from 30 to 100, and a Shore OO scale ranging from 0 to 100. For the validation of measuring reliability in scale A, some specimens had to be tested on a durometer calibrated outside of the laboratory.

Table 1 contains listed hardness and their standard deviation for all the materials in both scales.

For each material, five hardness measurements were performed and the mean value was used for the correlation model; the standard deviation did not exceed 1.8 Shore units for all materials on both scales.

## 3. Results

The search for the approximation function that best fits the calculated data was set to meet a few criteria: a minimum number of members (base functions), a minimum number of sections in the case of piecewise functions, the smallest *S*_2_ and the smallest maximum value of absolute relative error, a non-zero positive value of the elastic modulus at a hardness value of 0, a “reasonable” at a hardness value of 100, and the overall monotonicity. The procedure performed in the search set up the algorithm for the determination of the unknown parameters, calculating them based on the interpolation conditions in chosen data points, and calculating all the data for the criteria set, i.e., the *S*_2_, the maximum value of absolute relative error, and the standard deviation. In the following sections, details of approximation functions that best fit the data in both hardness scales are presented.

### 3.1. The Correlation of Elastic Modulus on the Shore OO Scale

**Figure 14:**
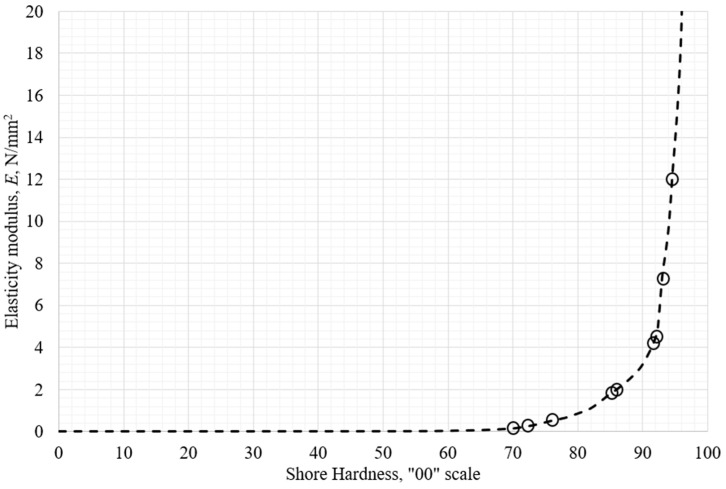
Measured data of modulus as a function of hardness OO scale approximated (shown) by piecewise polynomials.

After the conducted measurements as described in the previous sections, a diagram of the initial tensile elastic modulus as a function of Shore hardness on the OO scale approximated (graphically shown) in MS Office Excel is shown in Figure 14. The circles represent measured data points.

The first choice of the approximation function was a polynomial defined on the entire hardness scale in the form of:

(7) $$E_{\mathrm{app}}=\sum_{i=0}^{n}a_{n}{\left(H_{\mathrm{OO}}\right)}^{n}.$$

**Figure 15:**
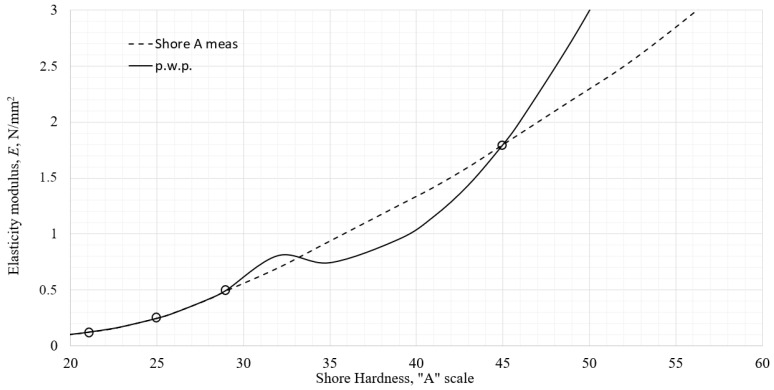
Loss of monotonicity by interpolation of the measured data using the discarded piecewise polynomial approximation function.

In Equation (7), *a_n_* is the unknown coefficient. Testing the polynomials up to degree 30 resulted in loss of monotonicity, and in some cases negative values for modulus; therefore, they were discarded. For brevity’s sake, only one figure of the problems encountered in various functions is shown here to depict what a discarded function gives as the correlation between the hardness and elastic modulus. In Figure 15, a piecewise polynomial function is shown to be representative of all the discarded functions in both hardness scales. In that particular case, the first part of the polynomial is defined on the hardness section ranging from 0 ≤ *H* ≤ 29 Shore A, and the second polynomial is defined on the hardness section ranging from 29 ≤ *H* ≤ 66 Shore A, the choice of which is guided by the available data points and the apparent curvature of the curve approximated between the data points. This provides the reader with an explanation of the types of problems that arise when approximating the elastic modulus as a function of hardness with polynomial and rational functions, and a sum of exponentials defined on the whole hardness scale. The rational function also demonstrated similar problems, which is dependent on the choice of degree and in which points interpolation conditions were enforced. Least squares approximations resulted in even less control over the error.

The rational function tested in this manuscript was composed from polynomial functions, chosen with different degrees in the numerator and denominator. The reason for this is the search for the minimal degree of function firstly in numerator polynomial, such that with a reasonable degree of denominator polynomial, the *S*_2_ and the maximum value of the relative error would be minimal. The degree of the denominator will be determined by enforcing the interpolation in chosen data points. Some of the tested functions have a form described in the following equation:

(8) $$\begin{array}{l} E_{\mathrm{app}}=\frac{a_{0}+a_{1}H_{\mathrm{OO}}+a_{2}{\left(H_{\mathrm{OO}}\right)}^{2}+a_{3}{\left(H_{\mathrm{OO}}\right)}^{3}}{b_{0}+b_{1}H_{\mathrm{OO}}+b_{2}{\left(H_{\mathrm{OO}}\right)}^{2}+b_{3}{\left(H_{\mathrm{OO}}\right)}^{3}+b_{4}{\left(H_{\mathrm{OO}}\right)}^{4}},\\ E_{\mathrm{app}}=\frac{a_{0}+a_{1}{\left(H_{\mathrm{OO}}\right)}^{10}+a_{2}{\left(H_{\mathrm{OO}}\right)}^{20}+a_{3}{\left(H_{\mathrm{OO}}\right)}^{30}}{b_{0}+b_{1}H_{\mathrm{OO}}+b_{2}{\left(H_{\mathrm{OO}}\right)}^{2}+b_{3}{\left(H_{\mathrm{OO}}\right)}^{3}+b_{4}{\left(H_{\mathrm{OO}}\right)}^{4}}\;. \end{array}$$

The first function in Equation (8) is an attempt with a low degree function, while the second is one of the tested ones with a high degree. Quality of approximation functions is verified by both calculating the *S*_2_ error norm [6]:

(9) $$S_{2}=\sum_{i=1}^{10}{\left(\frac{E_{i,\mathrm{meas}}-E_{\mathrm{app}}\left(H_{\mathrm{OO}i}\right)}{E_{i,\mathrm{meas}}}\right)}^{2}\to \min,$$

and minimizing the maximum value of absolute relative error in the form of:

(10) $$\left|\frac{E_{i,\mathrm{meas}}\left(H_{\mathrm{OO}i}\right)-E_{\mathrm{app}}}{E_{i,\mathrm{meas}}\left(H_{\mathrm{OO}i}\right)}\right|\to \min,\;i=1,10.$$

The search for the minimum *S*_2_ for the rational function is contained in the search for coefficients in the denominator, with preset coefficients in the numerator function. The aim is to choose the numerator polynomial closest to the approximating measured data, so that the denominator “corrects” that approximation to minimize the *S*_2_. Both of those functions exhibit fluctuation of error unacceptable for correlation, similar as that shown in Figure 15.

The sum of exponential functions seemed a promising approximation model, with preset parameters in the arguments. The tested form of such function is:

(11) $$\begin{array}{c} E_{\mathrm{app}}\left(H_{\mathrm{OO}}\right)=A\exp\left(H_{\mathrm{OO}}K_{1}^{\mathrm{OO}}\right)+B\exp\left(H_{\mathrm{OO}}K_{2}^{\mathrm{OO}}\right)+C\exp\left(H_{\mathrm{OO}}K_{3}^{\mathrm{OO}}\right)+\\ D\exp\left(H_{\mathrm{OO}}K_{4}^{\mathrm{OO}}\right)+J\exp\left(H_{\mathrm{OO}}K_{5}^{\mathrm{OO}}\right). \end{array}$$

The parameter $K_{i}^{\mathrm{OO}}$ in the exponential function (11) was preset in the interpolation procedure, variated along with variation in the choice of the data points for the interpolation condition. The coefficients *A* through *D* in (11) were determined for each selected set of parameters and interpolation points. The best fitted function resulted in a maximum value of the relative error of 22%, with all the criteria for the approximation function mentioned above. That was decided to be too large of an error.

**Figure 16:**
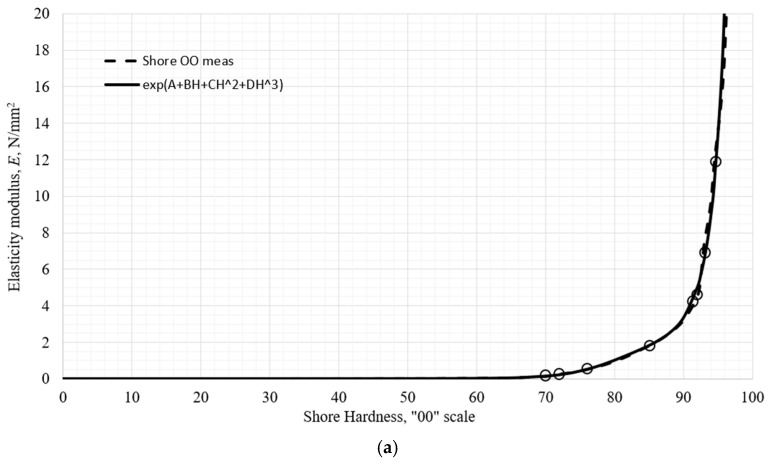

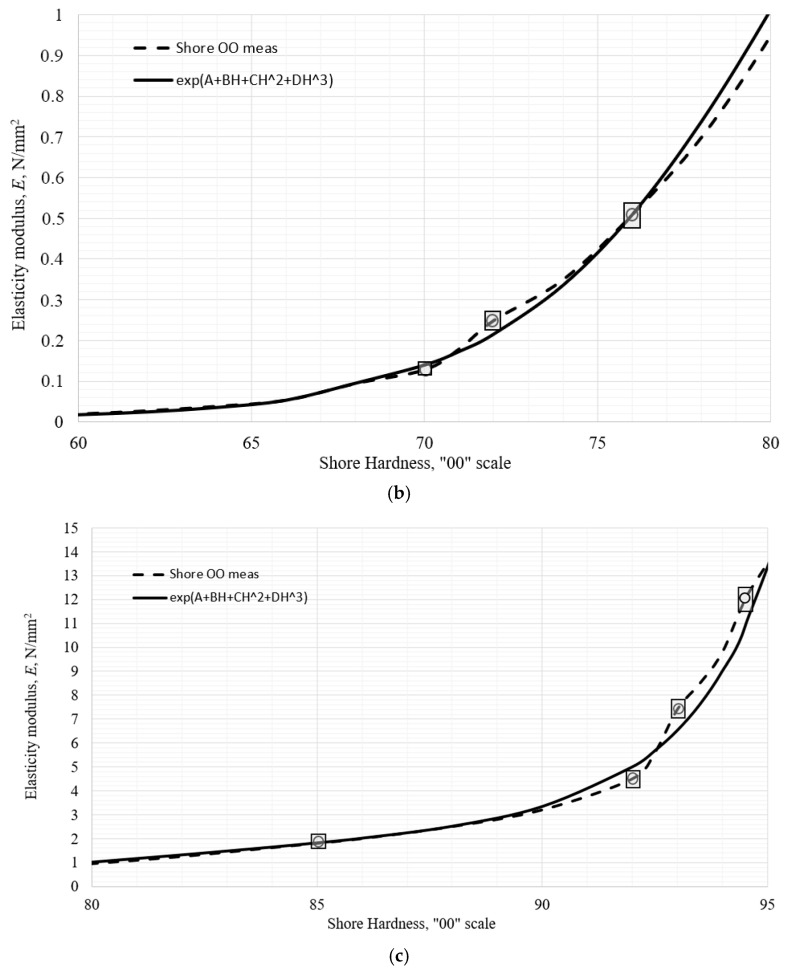
The elastic modulus of the tested materials approximated with a function of Shore OO hardness: (**a**) on the entire scale, (**b**) a detailed portion of the scale up to 80 Shore OO, (**c**) a detailed portion of the scale above 80 Shore OO.

The final form of the exponential function for testing as a piecewise exponential function was chosen, with its argument derived from the natural logarithm of elastic modulus in the hardness scale section. That data was approximated by a polynomial of degree three or four, depending on the quality of approximation. The general form of such an argument function is $f_{i}\left(H\right)=A_{i}+B_{i}H+C_{i}H^{2}+D_{i}H^{3}+\left(F_{i}H^{4}\right)$ for the *i*-th section of the scale. The final approximation of the elastic modulus is then calculated as $E_{\mathrm{app},i}\left(H\right)=\exp\left[f_{i}\left(H\right)\right].$ The “nature” of the data on the OO scale required division into two sections, the first ranging from 0 ≤ *H* ≤ 66 Shore OO, and the second one ranging from 66 ≤ *H* ≤ 100 Shore OO. The final correlation function is:

(12) $$E_{\mathrm{app}}\left(H\right)=\left\{\begin{array}{c} \exp\left[\begin{array}{l} -9.994+0.2716H-6.574\cdot 10^{-3}H^{2}+\\ 6.186\cdot 10^{-5}H^{3} \end{array}\right],\;0\leq H\leq 66\\ \exp\left[\begin{array}{l} 1049.59-54.925H+1.064H^{2}-\\ 9.064\cdot 10^{-3}H^{3}+2.878\cdot 10^{-5}H^{4} \end{array}\right],\;66\leq H\leq 100 \end{array}\right\}\mathrm{MPa},$$

resulting in *S*_2_ = 0.062, and the standard deviation s = 0.101. The approximated dependence of the modulus as a function of Shore OO hardness is shown in Figure 16. Vertical bars represent predicted upper and lower limits of the measured data for the modulus described previously. The full line represents the correlation function in (12).

An important characteristic of the function (12) is non-negativity at a hardness value of 0 and a “finite” value for the elastic modulus at a hardness value of 100 and is monotonic. The two curves at the joining point of 66 Shore OO shows a slight difference in slope, which does not affect the correlation significantly. The maximum relative error that function has at all the measured data points is 13.4% at 72 Shore OO, visible in Figure 16b. The two curves possess a C0 continuity.

### 3.2. The Correlation of the Elastic Modulus on the Shore A Scale

**Figure 17:**
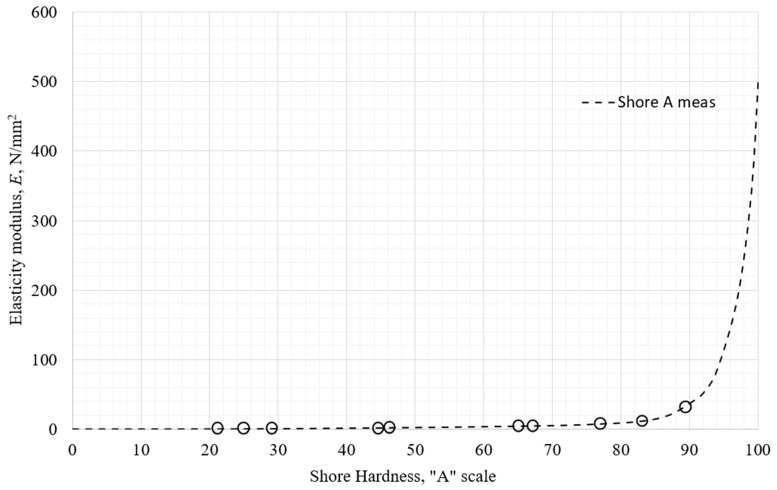
The elastic modulus of the tested materials as a function of Shore A hardness.

After the conducted measurements in the previous sections, a diagram of the initial tensile elastic modulus as a function of Shore hardness on the A scale approximated (graphically shown) in MS Office Excel is shown in Figure 17. The circles represent measured data points.

The search for the optimum approximation function on the Shore A scale followed a similar path and problems as the one on the Shore OO scale, detailed above. The sum of exponential functions similar to that in (11) resulted in too large a relative error of 60%. In most of the hardness ranges, it correlated with a relative error of around 20% or less. For this reason, it was discarded.

**Figure 18:**
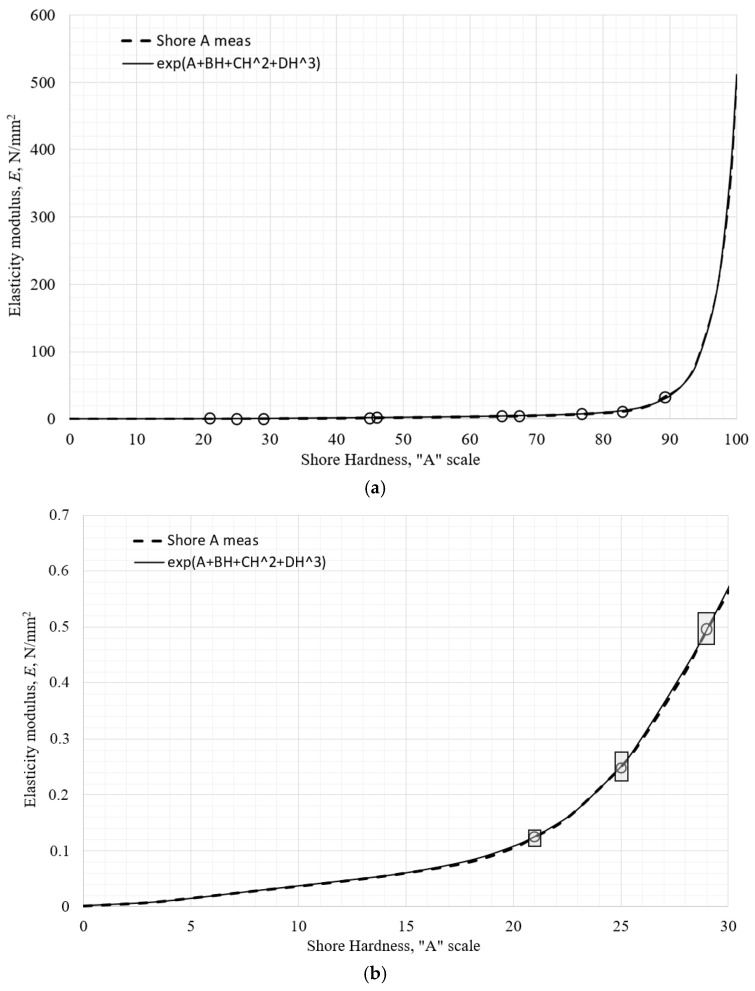

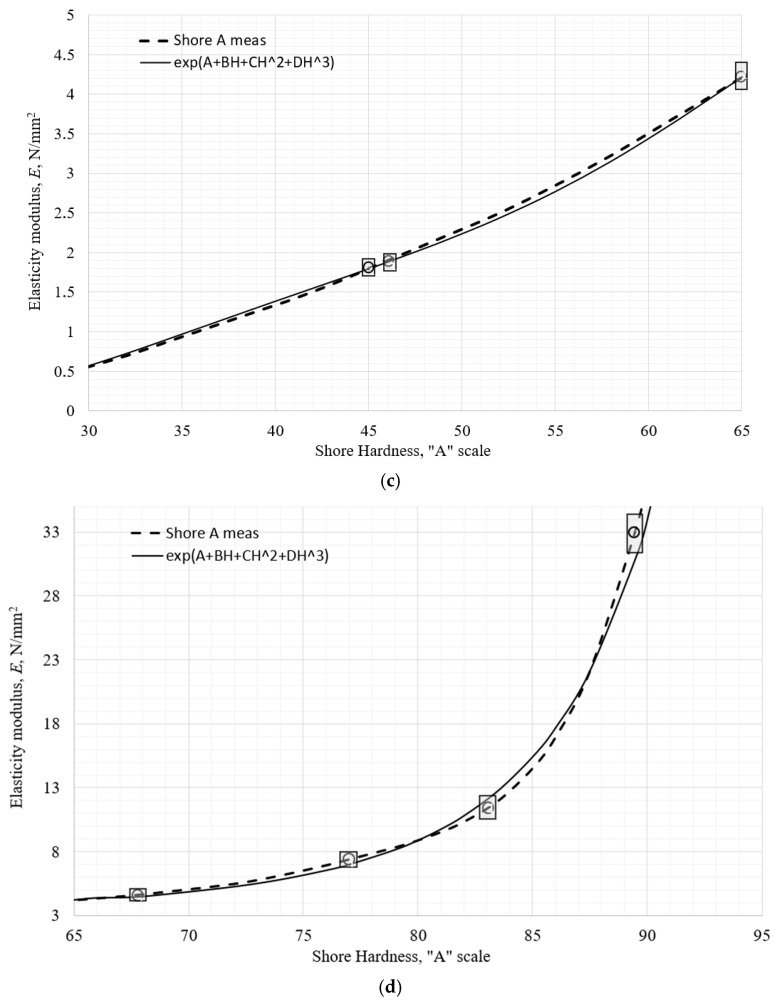
The elastic modulus of the tested materials approximated as a function of Shore A hardness: (**a**) in whole scale, (**b**) detailed portion of scale below 30 Shore A, (**c**) detailed portion of scale between 30 and 65 Shore A, (**d**) detailed portion of scale above 65 Shore A.

The final approximation function was chosen as a piecewise exponential function with its argument derived from the natural logarithm of the elastic modulus in the chosen hardness scale section, the same as on the OO scale. That data was approximated by a polynomial of degree three or four, depending on the quality of approximation. The “nature” of the data on the A scale required division into three sections, the first ranging from 0 ≤ *H* ≤ 25 Shore A, the second ranging from 25 ≤ *H* ≤ 67 Shore A, and the third ranging from 67 ≤ *H* ≤ 100 Shore A. The final correlation function is:

(13) $$E_{\mathrm{app}}\left(H\right)=\left\{\begin{array}{c} \exp\left[\begin{array}{l} -6.637+0.7075H-5.578\cdot 10^{-2}H^{2}+\\ 2.115\cdot 10^{-3}H^{3}-2.719\cdot 10^{-5}H^{4} \end{array}\right],\;0\leq H\leq 25\\ \exp\left[\begin{array}{l} -15.18+1.072H-2.863\cdot 10^{-2}H^{2}+\\ 3.526\cdot 10^{-4}H^{3}-1.623\cdot 10^{-6}H^{4} \end{array}\right],\;25\leq H\leq 67\\ \exp\left[\begin{array}{l} -18.869+0.903H-1.383\cdot 10^{-2}H^{2}+\\ 7.529\cdot 10^{-5}H^{3} \end{array}\right],\;67\leq H\leq 100 \end{array}\right\}\mathrm{MPa},$$

resulting in *S*_2_ = 0.013 7, and standard deviation s = 0. 013. The correlation function is plotted against the approximated measured data in Figure 18a. The vertical bars in Figure 18b–d represent predicted limits of the measured data as described previously.

The joining points at 25 Shore A and 67 Shore A show practically no difference in slope, although that continuity condition was not enforced. The maximum relative error that function exhibits at all the measured data points is 7% at 89.5 Shore A, visible in Figure 18d.

## 4. Discussion

The results of this study confirm a strongly nonlinear relationship between Shore hardness and the initial elastic modulus of flexible polymers, consistent with classical and recent investigations. The use of the true stress and logarithmic (Hencky) strain [4], combined with iterative correction of the cross-sectional area, provides a physically consistent representation of the initial tangent modulus and improves upon conventional engineering strain approaches often applied in elastomer characterization. This approach aligns with prior recommendations for accurate modeling of large strain behavior in soft materials [4].

Gent’s classical theoretical work [10] established the foundational relation between the indentation hardness and elastic modulus for elastomers, but also highlighted that simplified analytical relations lose accuracy at low hardness values and in regimes dominated by large deformations. Our results demonstrate similar limitations: polynomial correlations may approximate mid-range hardness behavior, but they systematically fail to capture the rapid modulus escalation observed near the upper end of both Shore scales.

Meththananda et al. [11] examined dental elastomers and reported a generally strong correlation between Shore hardness and the elastic modulus, yet they also observed significant deviations at both low and high hardness values, where empirical adjustments become required [11]. Our findings on silicone rubbers, silicone, TPU, and NBR materials confirm this behavior, particularly in the Shore OO range, where a sharp increase in modulus is observed above ~85 Shore OO. This supports the use of advanced nonlinear empirical models capable of capturing this transition [12,13].

The sum of exponentials correlation developed in this work proved superior to polynomial and rational functions. It preserved monotonicity, ensured positive values at *H* = 0, and yielded finite modulus predictions at *H* = 100. These properties match physical reality recommendations from industrial and computational studies, which emphasize that hardness–modulus relations in elastomers are best described using nonlinear, frequently exponential-type mappings [9,12,14]. However, in the Shore A scale, the maximum relative error was 60%, and in the Shore OO scale around 22%. For this reason, that model was discarded.

The final developed correlation model is a piecewise exponential function, with its argument in the form of polynomial function derived using the approximation of natural logarithm of the elastic modulus data. The polynomial function has degree three or four, depending on the achieved approximation quality. The hardness scale sections in which individual correlation function is defined was chosen according to the approximation quality and the trend of data change, i.e., the rate of change in the elastic modulus with the increasing hardness. Each hardness section has one exponential function for the correlation. Finally, for the Shore A scale, three sections were necessary to achieve acceptable correlation quality, while in the Shore OO scale, two sections were necessary. That model predicts initial modulus reliably within the experimental scatter (≈±8%) and with prediction errors below ~13.4% on the OO scale, and ~7% in the A scale.

Recent polymer studies also highlight the influence of microstructural parameters—crosslink density, filler distribution, and viscoelasticity on the hardness–modulus relationship [13]. Further testing the piecewise approximation, and expanding the model to incorporate dynamic or time-dependent behavior remains a promising avenue for future research.

Overall, the proposed piecewise exponential correlation aligns with classical theory [10], established empirical findings [11], and modern polymer mechanics research [9,13,15,16], offering a robust and practically useful tool for estimating the modulus from the hardness measurements in flexible polymer systems.

## 5. Conclusions

The manuscript offers a new mathematical model for the correlation between the Shore hardness of flexible polymers in the A and OO scales and the initial tensile elastic modulus. The correlation model consists of piecewise exponential functions that possess C0 continuity. Arguments for those functions were derived using approximation by polynomials of the natural logarithm of the elastic modulus data.

The materials tested in this contribution are flexible polymers, namely, silicone rubbers, nitrile butadiene rubbers (NBR), silicone, and thermoplastic polyurethane (TPU). All modulus values reported in this study were calculated with a maximum error of 8% relative to the mean value. This includes the force value scatter and cross-sectional measurements scatter. The scatter is shown in the diagrams as bars of min–max value at the measured points. For most of the tested materials, the correlation was derived on both scales, with TPU 98A being the only one with a hardness value over 100 on the OO scale.

The stress was calculated as the current force divided by current cross-sectional area, with the strain calculated using a logarithmic equation. Many of the tested materials exhibit pronounced hyperelastic behavior, so the initial modulus (slope) from their stress–strain curves was taken for the correlation model. Five specimens were tested for each material. In the correlation, the mean value of hardness from five measurements in both scales was taken as the representative for the material, but the scatter was not introduced into the model. The scatter of hardness in both scales did not exceed 1.8 units of hardness.

Three types of functions were tested for the correlation of the modulus as a function of the hardness in both the A and OO scales. The discarded functions are the polynomial, the rational function with polynomials of various degrees in the numerator and denominator, and the sum of exponential function. Although polynomial and rational functions can provide acceptable interpolation within limited hardness ranges, they exhibit undesirable behavior at the boundaries of the Shore scales, including loss of monotonicity and unrealistic extrapolation near *H* = 0 and *H* = 100. In contrast, the sum of exponentials model preserves physical consistency by ensuring strictly positive modulus values and finite asymptotic behavior, while maintaining a lower global approximation error.

The new, developed model a reader can easily calculate, presented in this study, exhibits less than 13.4% relative error w.r.t. the measured modulus on the entire OO scale, and a maximum relative error less than 7% on the entire A scale.

One further consideration of improvement of the mathematical model, along with adding new materials in testing, is the introduction of the piecewise polynomials as the approximation function. The first choice is the C1 continuous cubic spline, and the second choice a B-spline of third degree with interpolation properties at chosen points of interest. Particular interest for further research is the development of a correlation model for bio-compatible polymers for dental application, with hardness on the Shore OO scale.

## Figures and Tables

**Table 1:** The Shore hardness of polymers tested.

Shore Hardness	Silicone Rubber 15 A	Silicone Rubber 20 A	Silicone Rubber 30 A	NBR 40A	NBR 45A	Silicone 60 A	TPU 60 A	TPU 70 A	TPU 85 A	TPU 98 A
OO scale	70	72	76	85	85.5	91.5	92	93	94.5	-
Standard deviation	1.11	1.04	1.2	1.15	1.55	1.27	1.68	1.44	1.78	-
A scale	21	25	29	45	46	65	67.5	77	83.5	89.5
Standard deviation	0.45	0.52	0.64	0.67	0.62	0.48	0.55	0.57	0.55	0.85

## Data Availability

The original contributions presented in this study are included in the article. Further inquiries can be directed to the corresponding author.

## Abbreviations

The following abbreviations are used in this manuscript:

NBR	Nitrile butadiene rubber
TPU	Thermoplastic polyurethane
FDM	Fused deposition modeling
w.r.t.	With respect to
